# Changes in Inpatient Coding for Hepatic Encephalopathy After Introduction of ICD‐10 Code K76.82

**DOI:** 10.1111/liv.70750

**Published:** 2026-06-18

**Authors:** Spencer R. Goble, Thomas M. Leventhal

**Affiliations:** ^1^ Division of Gastroenterology, Hepatology, and Nutrition, Department of Medicine University of Minnesota Minneapolis Minnesota USA

**Keywords:** hepatic encephalopathy (HE), hospitalization, inpatients, international classification of diseases

## Abstract

An ICD‐10 code specific for hepatic encephalopathy (HE), K76.82, was introduced in October of 2022. We aimed to assess changes in HE documentation following the introduction of this code. Using the National Inpatient Sample, we compared utilization of ICD‐10 codes historically used to identify HE before and after K76.82. From 2016 to 2021, 20.0% of cirrhosis hospitalizations included a non‐specific HE code, decreasing to 4.7% in 2023. K76.82 was used in 20.3% of hospitalizations in 2023. The introduction of K76.82 has dramatically changed the documentation of HE, and future studies assessing HE trends and outcomes need to account for these changes.

## Key Points

- Several non‐specific ICD‐10 codes have historically been used to identify hepatic encephalopathy.
- In October 2022, K76.82, an ICD‐10 code specific to hepatic encephalopathy was introduced.
- We found high utilization of K76.82 in 2023 with substantial decreases in the documentation of the previously used non‐specific codes.
- The adoption of K76.82 was associated with a substantial increase in the overall documentation of hepatic encephalopathy.
- Future studies assessing temporal trends in hepatic encephalopathy will need to account for the shift in documentation of hepatic encephalopathy caused by the introduction of K76.

**Abbreviations**
HE: hepatic encephalopathy
ICD‐10: International Classification of Diseases 10th Revision
NIS: National Inpatient Sample

## 1 Introduction

Hepatic encephalopathy (HE) is a major contributor to morbidity and mortality in patients with cirrhosis [1, 2, 3]. Historically, quantifying the burden and clinical impact of HE has been challenging due to the lack of a specific ICD‐10 (International Classification of Diseases 10th Revision) code and variable practices for identifying HE in administrative data sets that rely on ICD‐10 coding [4]. In October 2022, a specific ICD‐10 code for HE (K76.82) was introduced. Early assessment of K76.82 has demonstrated promising sensitivity and specificity [5]. However, there has been no large‐scale, national analysis of K76.82 utilization or how the use of previously employed codes to identify HE has changed following its implementation. Understanding these changes is critical, as any study evaluating trends or outcomes in HE must account for evolving coding practices to generate accurate and meaningful results. To address this, we assessed the utilization of K76.82 in 2023 using the National Inpatient Sample (NIS) and analysed trends in the utilization of other ICD‐10 codes historically used to identify HE before and after the introduction of K76.82.

## 2 Methods

This retrospective analysis used the NIS for years 2016–2021 and 2023. All hospitalizations from 2022 were excluded to avoid capturing admissions during the early implementation period of K76.82. The NIS is an administrative inpatient database that includes approximately 20% of all hospitalizations in the United States that are selected via a systematic stratified sampling design [6]. The Healthcare Cost and Utilization Project provides standardized methods for weighting the NIS data. When weighting is applied, as it was in this study, results are then intended to represent the entire United States inpatient population [6, 7].

Hospitalizations during the assessed years were included if the patient had a primary or secondary diagnosis of cirrhosis, defined using ICD‐10 codes. In the NIS, the primary diagnosis represents the condition chiefly responsible for the admission. Rates of utilization for ICD‐10 codes used to identify HE were assessed before (2016–2021) and after (2023) implementation of K76.82 among all hospitalizations involving patients with cirrhosis. Annual United States population estimates were obtained from the United States Census Bureau and used to calculate hospitalization rates per 10 000 population [8]. Based on prior studies, the following codes were included as indicators of HE: K72.90, K72.91, K72.01, K72.11, G93.40 and G93.49 [4, 9]. The following code groupings were also assessed: K72.90, K72.91, K72.01 and K72.11; and K72.90, K72.91, K72.01, K72.11, G93.40 and G93.49.

## 3 Results

**FIGURE 1:**
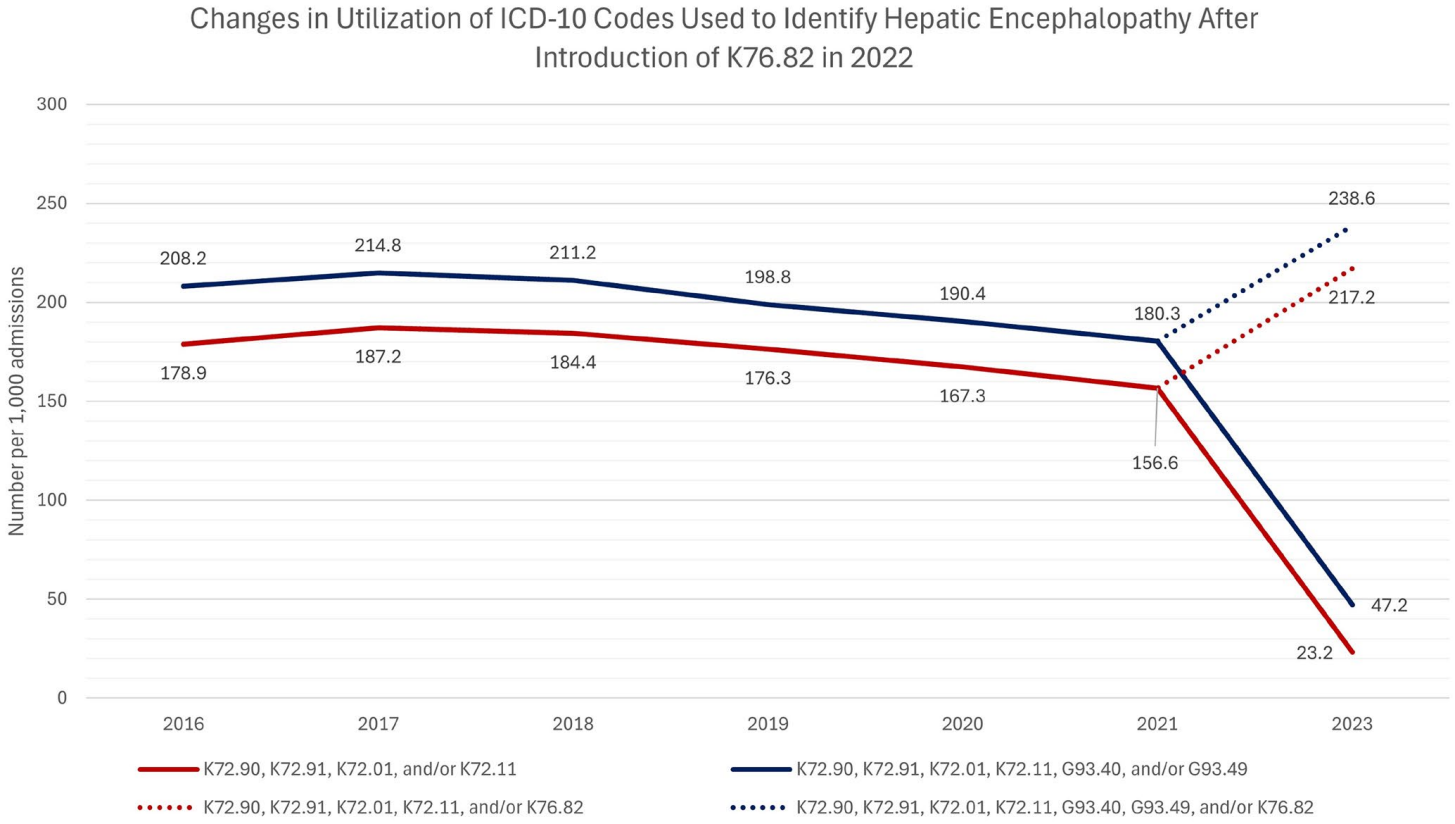
Documentation of ICD‐10 codes used to identify hepatic encephalopathy in hospitalized patients with cirrhosis before and after introduction of K76.82 in 2022.

A total of 5 505 888 hospitalizations involving patients with cirrhosis were identified. From 2016 to 2021, the most commonly documented ICD‐10 code was K72.90, present in 17.1% of admissions and listed as the primary diagnosis in 4.8%. One or more of the assessed ICD‐10 codes was present in 20.0% of admissions during this period and accounted for 5.1% of primary diagnoses. Utilization of these codes peaked in 2017, when 21.5% of admissions included at least one of the assessed diagnoses, and declined in each subsequent year to 18.0% in 2021. Following implementation of K76.82, there was a steep decline in the use of these codes, with only 4.7% of admissions including one of them in 2023 (Figure 1), representing a 73.8% decline in utilization from 2021 to 2023.

In 2023, 20.3% of admissions involving patients with cirrhosis had a diagnosis of K76.82 (*n* = 187 560), and 5.6% of admissions listed K76.82 as the primary diagnosis. Another evaluated HE‐associated code was present in 5.5% of admissions in which K76.82 was documented. Among admissions without K76.82, another evaluated code was present in 4.5% of cases. When K76.82 and all other assessed codes were considered together, HE was documented in 23.9% of admissions and listed as the primary diagnosis in 5.9% of admissions. After inclusion of K76.82, 2023 demonstrated the highest overall rate of HE‐associated coding, representing a 32.3% increase in documented cases compared with 2021. There were 6.60 hospitalizations per 10 000 United States population in 2023 when all codes, including K76.82, were included (Table 1). The previous highest rate was 4.88 per 10 000 in 2018.

**TABLE 1:** Number of hospitalizations with ICD‐10 documentation of hepatic encephalopathy per 10 000 united states population.

Year	US population	Rate per 10 000 United States population			
		K72.90, K72.91, K72.01 and/or K72.11	K72.90, K72.91, K72.01, K72.11 and/or K76.82	K72.90, K72.91, K72.01, K72.11, G93.40 and/or G93.49	K72.90, K72.91, K72.01, K72.11, G93.40, G93.49 and/or K76.82
2016	323 127 513	3.79	—	4.41	—
2017	324 657 169	4.12	—	4.73	—
2018	326 971 407	4.26	—	4.88	—
2019	328 239 523	4.28	—	4.82	—
2020	331 449 281	3.97	—	4.52	—
2021	331 893 745	3.97	—	4.57	—
2023	334 914 895	0.64	6.01	1.30	6.60

## 4 Discussion

In this retrospective assessment of HE coding practices, we observed high utilization of K76.82 in 2023, accompanied by substantial declines in the use of other codes historically employed to document HE. These findings suggest that K76.82 has largely replaced prior codes, with utilization more than fourfold higher than that of all other assessed codes combined in 2023. Additionally, the widespread adoption of K76.82 was associated with a marked increase in overall documentation of HE.

These findings have important implications for future research evaluating HE using ICD‐10–based data. Analyses of temporal trends in HE must account for these changes in coding practices, as attempts to assess longitudinal burden may be substantially confounded by the observed shifts following implementation of K76.82. Cautious interpretation is also warranted for studies assessing outcomes over time. Although the NIS lacks clinical granularity, it is plausible that the clinical scenarios prompting use of K76.82 differ from those associated with previously used codes, which may influence comparisons across years.

Overall, our results demonstrate that implementation of K76.82 has significantly altered inpatient coding for HE among patients with cirrhosis. Future studies using ICD‐10 codes to evaluate HE must account for these changes to ensure valid interpretation of trends and outcomes.

## Funding

The authors have nothing to report.

## Conflicts of Interest

The authors declare no conflicts of interest.

## Data Availability

The data that support the findings of this study are available in the National Inpatient Sample at https://hcup‐us.ahrq.gov/nisoverview.jsp.
